# Genome-Wide Identification, Phylogenetic, and Expression Analysis of Jasmonate ZIM-Domain Gene Family in *Medicago Sativa* L.

**DOI:** 10.3390/ijms251910589

**Published:** 2024-10-01

**Authors:** Jing Cui, Xu Jiang, Yajing Li, Lili Zhang, Yangyang Zhang, Xue Wang, Fei He, Mingna Li, Tiejun Zhang, Junmei Kang

**Affiliations:** 1 Institute of Animal Science, Chinese Academy of Agricultural Sciences, Beijing 100193, China; cuijing0417@yeah.net (J.C.); jiangxu2009@yeah.net (X.J.); ly_yajing@yeah.net (Y.L.); lili_chang721@yeah.net (L.Z.); yang20170115@163.com (Y.Z.); wangxue01@caas.cn (X.W.); hefei0609@126.com (F.H.); limingna@caas.cn (M.L.); 2 School of Grassland Science, Beijing Forestry University, Beijing 100083, China

**Keywords:** alfalfa, *MsJAZ*, salt stress, genome-wide

## Abstract

JASMONATE ZIM domain (JAZ) proteins, inhibitors of the jasmonic acid (JA) signaling pathway, are identified in different plants, such as rice and *Arabidopsis*. These proteins are crucial for growth, development, and abiotic stress responses. However, limited information is available regarding the *JAZ* family in alfalfa. This study identified 11 *JAZ* genes (*MsJAZs*) in the “Zhongmu No.1” reference genome of alfalfa. The physical and chemical properties, chromosome localization, phylogenetic relationships, gene structure, *cis*-acting elements, and collinearity of the 11 *MsJAZ* genes were subsequently analyzed. Tissue-specific analysis revealed distinct functions of different *MsJAZ* genes in growth and development. The expression patterns of *MsJAZ* genes under salt stress conditions were validated using qRT-PCR. All *MsJAZ* genes responded to salt stress, with varying levels of upregulation over time, highlighting their role in stress responses. Furthermore, heterogeneous expression of *MsJAZ1* in *Arabidopsis* resulted in significantly lower seed germination and survival rates in OE-2 and OE-4 compared to the WT under 150 mM NaCl treatment. This study establishes a foundation for further exploration of the function of the *JAZ* family and provides significant insights into the genetic improvement of alfalfa.

## 1. Introduction

Plants adapt to many stimuli during growth and development, including biotic stress, such as pests and diseases, as well as abiotic stress, such as salt, cold, and drought [1,2,3]. Phytohormones, such as auxins, cytokinins, ethylene, gibberellins, brassinosteroids, and jasmonates, are crucial for regulating physiological and molecular responses to abiotic stresses [4,5]. Among these, jasmonic acid (JA) is a vital signaling molecule. It regulates a wide range of cellular activities [6]. JA and its cyclic precursors and derivatives are collectively known as jasmonates (JAs). They play important roles in regulating various physiological processes in plants, including growth, development, and defense [7,8]. Jasmonate ZIM domain proteins (JAZ) can mediate the switch-like activation and repression of the JA pathway [9].

JAZ proteins are a subfamily of the TIFY superfamily and possess two conserved domains: the TIFY domain, characterized by the amino acid pattern of TIF [F/Y] XG, and the Jas domain, with a characteristic motif of SLX_2_FX_2_KRX_2_RX_5_PY [10,11]. JAZ is a negative regulator of the JA signaling pathway [12]. The JA content in plants is dynamically regulated [13]. Under normal conditions, JAs are present at low levels, and JAZ proteins inhibit the activity of many transcription factors, such as MYC2, MYC3, and MYC4 [9]. If the JA level is high, JAZ proteins will interact with JA-Ile receptor complexes, promoting the formation of co-receptor complexes consisting of the F-box protein COI1 and degron sequences located in the Jas motifs of JAZ. The hormone-dependent interaction of the COI1-JAZ co-receptor complex leads to JAZ protein degradation through the ubiquitin/26 s proteasome pathway, thereby releasing the JAZ-repressed expression of transcription factors, and many JA response genes are induced to express [14,15,16].

Previous studies have indicated that JAZ proteins are crucial in different growth processes in plants, including root growth, seed germination, and defense [17,18,19,20]. For instance, JAZ proteins can regulate jasmonate-enhanced root growth inhibition by inhibiting RHD6 and RHD6 LIKE1 transcription factors [17]. *OsJAZ1* negatively regulates development by repressing the bHLH-MYB complex. However, when the JAZ protein is degraded, it releases the bHLH-MYB complex, and activates downstream gene expression to regulate stamen development [21]. The ABA signaling pathway is vital for seed germination [22]. Studies have shown that the accumulation of JAZ proteins can attenuate ABA signaling, whereas *JAZ* mutants can enhance ABA responses [23]. Further analysis revealed that JAZ proteins repress the transcription of *ABI5* and *ABI3*, thereby regulating the ABA-mediated seed germination process [24]. Angiosperms can resist various stresses in nature through the COI1-JAZ-DELLA-PIF signaling pattern [25]. *JAZ* genes not only participate in the growth process of plants, but also respond to some abiotic stresses, including salt and drought stress, which are significant factors impacting plant growth and development [26,27]. In rice, OsJAZ9 interacts with OsNINJA and OsbHLH to form a transcriptional regulatory complex that regulates salt tolerance [28]. *OsJAZ1* enhances drought sensitivity through the JA and ABA pathways [29]. *PeJAZ2* positively reduces plant sensitivity to drought through ABA-induced stomatal closure by H_2_O_2_ production [30].

Alfalfa (*Medicago sativa* L.) is an important forage crop worldwide, and is known as the “King of Forages” [31]. Alfalfa has high nutritional value, such as rich protein content, and many kinds of vitamins [32]. Mining excellent genes in alfalfa is crucial for improving *M. sativa* research at the theoretical level, screening, and breeding new varieties. Previously, 12 and 15 *JAZ* genes were found in *Arabidopsis thaliana* [33] and rice [10], respectively, and 275 *JAZ* genes in 29 soybean genomes [34]. Despite its importance, the *JAZ* gene family in alfalfa remains underexplored. To address this gap, our study aimed to identify and analyze the *JAZ* gene family in *M. sativa*. We identified 11 JAZ proteins in the alfalfa genome and conducted a comprehensive analysis of the characteristics of 11 *MsJAZ* genes, including chromosome localization, phylogenetic analysis, gene structure collinearity analysis, and *cis*-acting element analysis. The evolutionary relationships between *MsJAZs* and *A. thaliana*, *Medicago truncatula*, and *Glycine max* were analyzed. To further analyze the potential function of *MsJAZs*, qRT-PCR was used to detect their expression patterns under salt stress. *MsJAZ1* was cloned and heterologously expressed in *Arabidopsis*, and the phenotype of transgenic *Arabidopsis* under salt stress was investigated. In summary, we identified and analyzed all the *JAZ* genes in the alfalfa genome, providing reference for further functional studies.

## 2. Results

### 2.1. Identification of JAZ Genes in the Alfalfa Genome

Eleven *JAZ* were identified in the *M. sativa* genome (Zhongmu No.1) through the hidden Markov model (HMM) and domain analysis, and these members were named *MsJAZ1*–*MsJAZ11* based on their chromosomal locations. The basic information regarding *MsJAZ* is listed in Table 1. The length of the MsJAZ proteins ranged from 131 aa (MsJAZ2) to 337 aa (MsJAZ11), with a mean length of 240 aa. The MW of the MsJAZ proteins ranged from 15.09 (MsJAZ2) to 38.03 kDa (MsJAZ1). The pI of MsJAZ proteins was between 7.10 (MsJAZ3) and 9.56 (MsJAZ6). These proteins were alkaline (pI > 7.0). All *MsJAZs* were located in the nucleus by subcellular localization prediction, while *MsJAZ1* and *MsJAZ3* were also found in the cell membrane.

**Figure 1:**
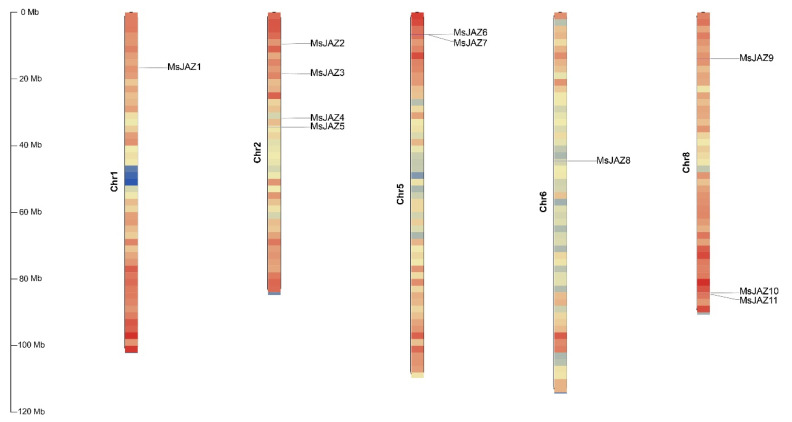
Chromosomal distribution diagram of alfalfa the *JAZ* gene. TBtools (v1.108, Chen, C., GZ, China) was used to visualize the position of each *MsJAZ* on the chromosome. Eleven *MsJAZs* are mapped onto the five chromosomes of *Medicago sativa*. The vertical bars represent the chromosomes of alfalfa. The scale on the left indicates the chromosome length (Mb). Color gradients indicate gene density, and red to blue indicate gene density from high to low. Each *MsJAZ* gene is labeled at its respective position on the chromosomes.

The 11 *JAZ* genes were distributed on 5 chromosomes of the alfalfa genome, among which no *MsJAZ* genes existed on chromosomes 3, 4, and 7 (Figure 1). The greatest number of *MsJAZ* genes (4 of 11) was distributed on chromosome 2, while three *MsJAZ* genes were located on chromosome 8. There are two *MsJAZ* genes on chromosome 5 and only one *MsJAZ* gene on chromosomes 1 or 6.

### 2.2. Phylogenetic Relationships Analysis of MsJAZs

**Figure 2:**
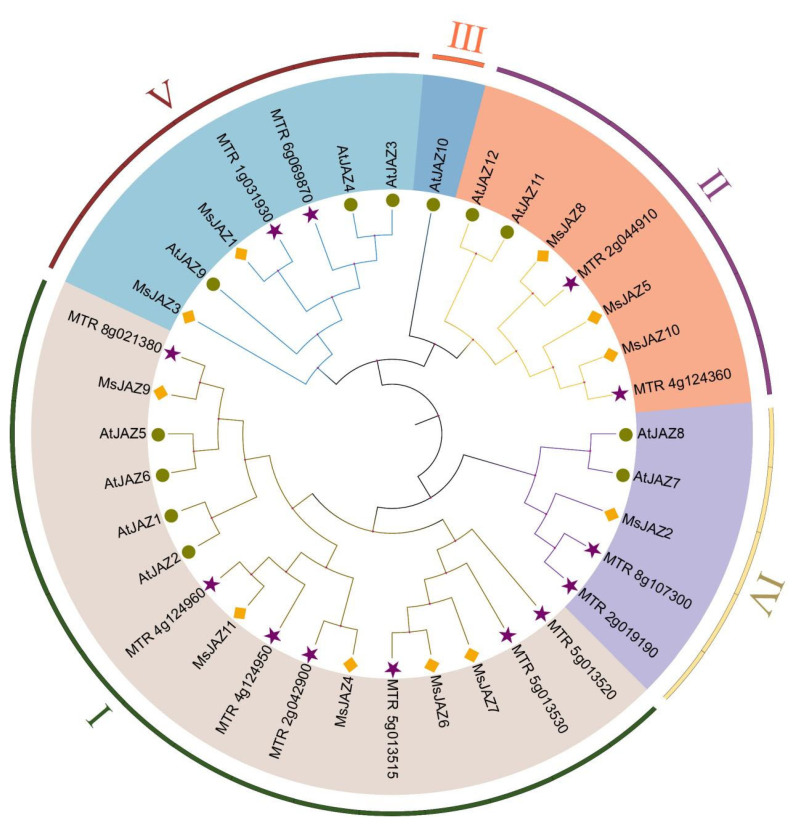
Phylogenetic tree of the *JAZ* genes in *M.sativa*, *M. truncatula*, and *A. thaliana.* The phylogenetic tree was constructed using the maximum likelihood method (bootstrap method with 1000 replications was selected) of MEGA 6.0 software. *JAZ* genes were divided into five groups (I–Ⅴ) according to the known *Arabidopsis JAZ* family, and were represented using different colors. Using different markers to distinguish each species, yellow diamonds, purple stars, and green circles, respectively, represent *M. sativa*, *M. truncatula*, and *A. thaliana*.

**Figure 3:**
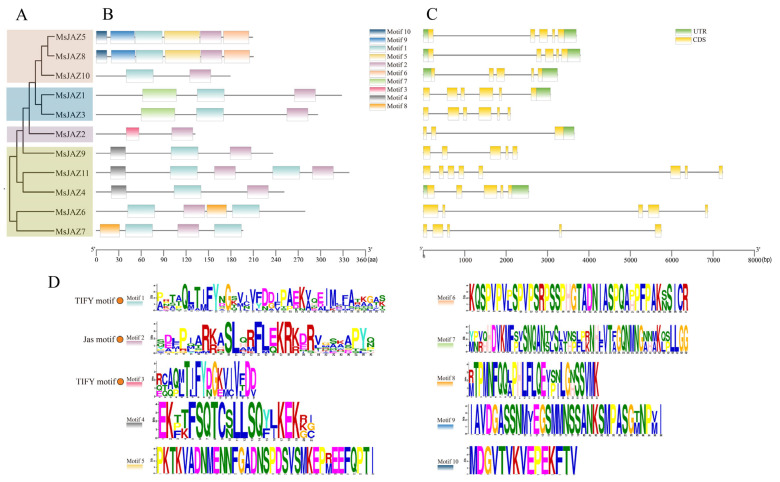
Phylogenetic relationships, motif compositions, and structures analysis of the MsJAZs family. (**A**) The phylogenetic tree of *MsJAZs* of *M. sativa* was constructed by the maximum likelihood method of MEGA 6.0 software. All *MsJAZs* were divided into four groups and represented by different colors. (**B**) The motif composition of *MsJAZs* was analyzed with the MEME program. A different color is used for each motif. (**C**) The UTR, CDS, and intron organization of *MsJAZs*. The green boxes, yellow boxes, and thin black lines represent UTRs, CDSs, and introns, respectively. (**D**) Sequence logos of motifs 1–10. Motif 1 and motif 3 represent the TIFY motif, and motif 2 represents the Jas motif.

To assess the evolutionary relationship of *JAZ* between alfalfa and other plants, a maximum likelihood phylogenic tree was generated, containing 11 in alfalfa, 12 in *A. thaliana*, and 13 in *M. truncatula* (Figure 2). Based on the known phylogenetic tree grouping of the *Arabidopsis JAZ* genes, the 36 JAZ proteins were divided into groups I–Ⅴ. Except for group Ⅲ, all other groups contained alfalfa *JAZ* family members. Group I included the most MsJAZ proteins, with five, accounting for 45% of the total, and group IV had the fewest MsJAZ proteins, with one. According to the grouping of phylogenetic trees, 11 MsJAZ were divided into four branches (Figure 3A), and their gene structure and motif composition were analyzed.

### 2.3. Gene Structure and Conserved Motif Analysis

The exon and intron structures of the *MsJAZ*s were investigated to understand the structural composition of these genes (Figure 3C). Genetic structure analysis revealed that *MsJAZ* genes exhibit some similarity in the four groups, but also present many differences between the groups. In total, 64% *MsJAZ* genes (7 of 11) have five exons. In group Ⅱ, all genes contained five exons, and had UTR sections at both terminals. In group Ⅴ, all genes had six exons. Only one gene (*MsJAZ2*) had the fewest number of exons in group Ⅳ, with three exons. In group I, *MsJAZ11* had eight exons, which was the largest number. All other genes contained five exons, and only *MsJAZ4* had UTR sections at both terminals.

In order to further analyze the function of MsJAZs, we analyzed the conserved motifs of 11 proteins. (Figure 3B). A total of ten motifs were detected, and the details of the ten motifs are provided in Table S2. The results showed that all MsJAZ proteins contained the TIFY (motif 1 or motif 3) and Jas motifs (motif 2) (Figure 3D) (Figure S1). Seven additional motifs were present only in specific groups. For example, motifs 5, 6, 9, and 10 were only discovered in group Ⅱ, motif 7 was only identified in group Ⅴ, and motifs 4 and 8 were only present in group Ⅰ. These results indicated that the *MsJAZ* of the same group had similar gene structures and motif compositions, whereas the *MsJAZ* in different groups possessed distinct structures and motifs, which might have contributed to the functional diversity among *MsJAZs*.

### 2.4. Prediction of Cis-Acting Elements in MsJAZs Promoters

**Figure 4:**
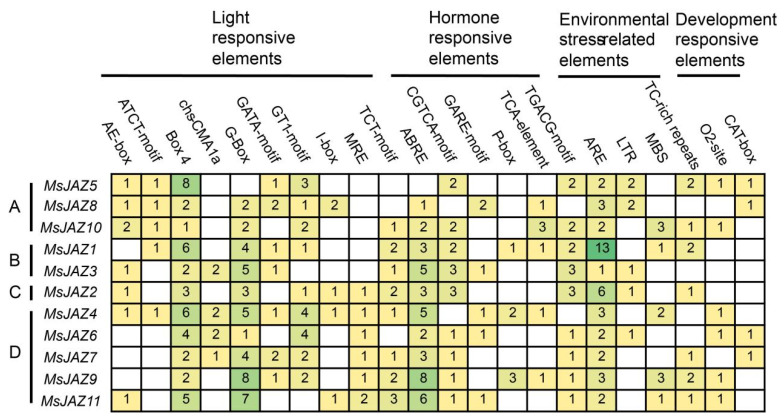
Prediction of *cis*-acting elements of *MsJAZ* genes. (A–D): *MsJAZ* genes were divided into four groups. *Cis*-acting elements are divided into light, hormones, environmental stress, and development response elements. The digit represents the number of elements.

To further investigate the potential functions of *MsJAZs*, the *cis*-elements in the promoter regions of the *MsJAZs* were predicted by PlantCARE. These *cis*-acting elements are divided into four categories: light, hormone, environmental stress, and development-responsive elements (Figure 4). Light-responsive elements mainly contain Box 4, GT1-motif, and G-box, and all genes contain Box 4 elements. Ten *MsJAZ* genes contained ABA response elements (ABRE), nine *MsJAZ* genes contained JA response elements (CGTCA and TGACG motifs), and five *MsJAZ* genes contained SA response elements (TCA-element). These hormone-responsive elements are crucial for plant biotic and abiotic stress resistance. In addition, several environmental stress-related elements were identified as being responsive to anaerobic conditions (ARE), low temperature (LTR), drought (MBS), and stress (TC-rich repeats). All *MsJAZ* genes contained ARE elements, with some numbers as high as 13. These results showed that *MsJAZ* genes have *cis*-acting elements of different types and numbers in their promoter regions, which may be related to their special functions.

### 2.5. Analysis of the Gene Duplication and Synteny of the MsJAZs

**Figure 5:**
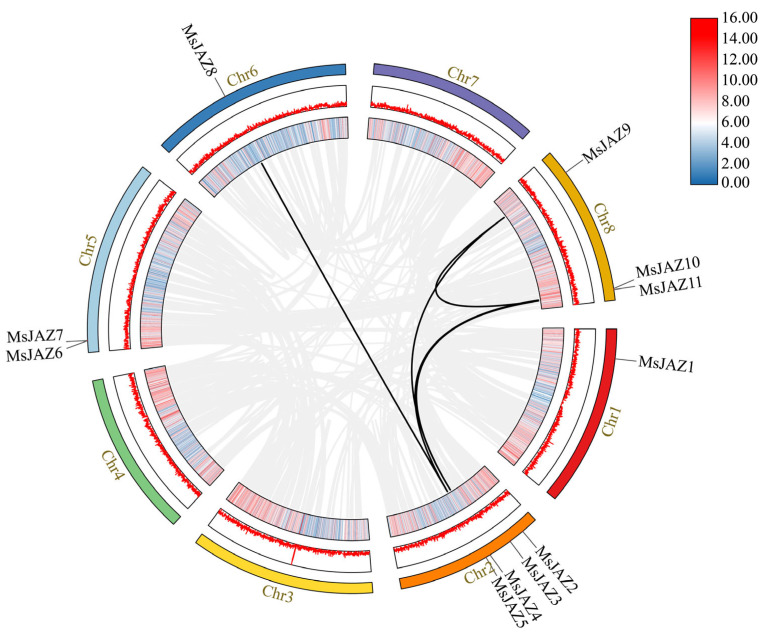
Schematic diagram of the syntenic relationships of *MsJAZ* genes in *M. sativa*. The outermost colored circles represent the eight chromosomes of alfalfa, and the position of the *MsJAZ* genes on the chromosome is marked on the circle. The gray lines show the syntenic regions in alfalfa genome, and the black lines indicate *JAZ* gene pairs in segmental duplication events. The innermost and the middle circles represent the density and abundance of genes at that location on the chromosome.

Genome duplication events in this family were investigated to understand the evolution of *MsJAZ* genes better. A syntenic relationship between the *MsJAZ* genes was discovered using MCScanX from TBTools. Five segmental duplication events were found in the *MsJAZs* (Figure 5). This result indicates that segmental duplication plays a crucial role in the evolution of the *MsJAZs* gene family. To better understand the evolutionary constraints, the Ka, Ks, and Ka/Ks ratios of the *MsJAZ* gene pairs were calculated. All five gene pairs had Ka/Ks ratios below 1, suggesting that the *MsJAZ* gene family experienced purification selection (Table 2).

**Figure 6:**
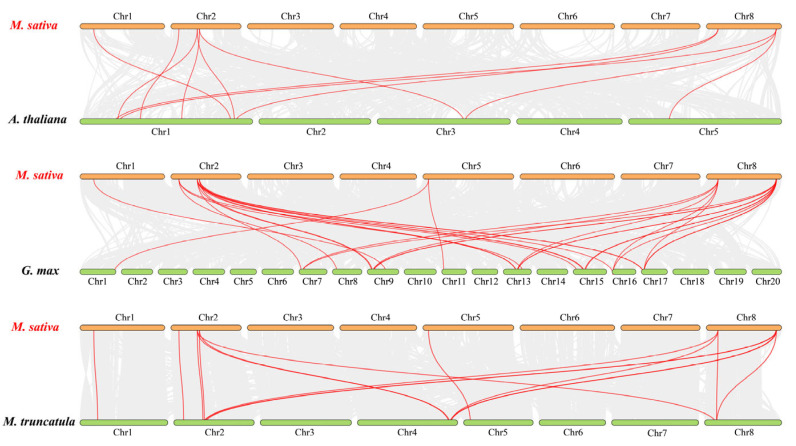
Synteny analysis of *JAZ* genes between *M. sativa* and three representative plant species. The orange boxes represent the chromosomes of *M. sativa*, and the green boxes represent the chromosomes of *A. thaliana*, *G. max*, and *M. truncatula*. Gray lines in the background indicate collinear blocks between alfalfa and the indicated plant, while the red lines indicate the duplication of *JAZ* gene pairs.

To further identify the orthologous relationships of *MsJAZ* genes in different crops, the synteny of *M. sativa* with *A. thaliana*, *G. max*, and *M. truncatula* was analyzed. The results showed that 11 orthologous gene pairs were identified between *Arabidopsis* and alfalfa, 16 between *M. truncatula* and alfalfa, and 31 between *Glycine max* and alfalfa (Figure 6). Seven *JAZ* genes (*MsJAZ1/2/4/5/9/10/11*) were simultaneously identified as orthologous genes between the three species and alfalfa genomes. These genes may play an essential role in the evolution of the *JAZ* family.

### 2.6. Expression Analysis of MsJAZ Genes in Different Tissues

**Figure 7:**
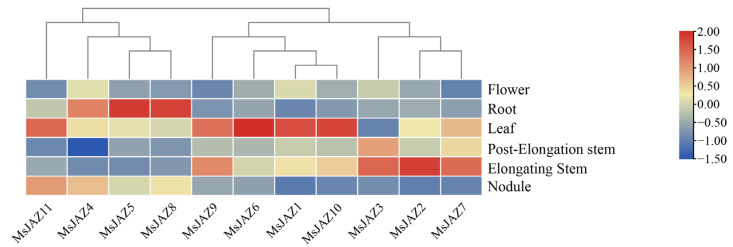
Expression analysis of *MsJAZ* genes in different tissues (flowers, roots, leaves, post-elongating stems, elongating stems, and nodules). The legend represents the relative expression levels, blue indicates low expression levels and red indicates high expression levels.

To further understand the expression patterns of *MsJAZ* genes in different tissues of alfalfa, transcriptome data of different tissues of alfalfa were obtained from public databases, including flowers, roots, leaves, post-elongation stems, elongating stems, and nodules (Figure 7). The original data are presented in Supplementary Table S1. In total, 45% *MsJAZ* genes (*MsJAZ1*, *MsJAZ6*, *MsJAZ9*, *MsJAZ10*, and *MsJAZ11*) were highly expressed in leaves, 27% (*MsJAZ3*, *MsJAZ2* and *MsJAZ7*) in the elongating stems, and 27% (*MsJAZ4*, *MsJAZ5* and *MsJAZ8*) in the roots. The expression levels of all *MsJAZ* genes were low in flowers. These results show that different *MsJAZ* genes may play different roles in regulating the growth and development of alfalfa.

### 2.7. Expression Analysis of MsJAZ Genes in Salt Stress Treatments

**Figure 8:**
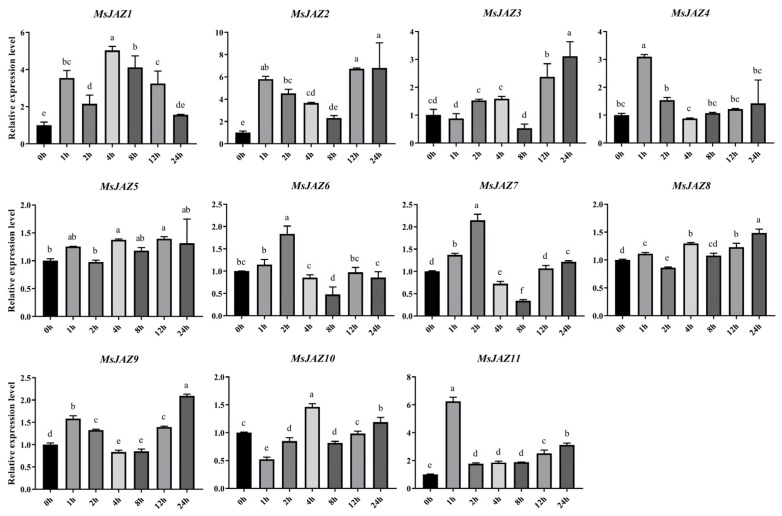
Expression patterns of 11 *MsJAZ* genes under 200 mM NaCl treatment. The horizontal coordinates represent processing times, and the vertical coordinates represent relative expression levels. The error bars represent the standard error of the means of three independent replicates. ANOVA with *p* < 0.05. There are significant differences between different letters (a–f).

To understand the response of the 11 *MsJAZs* to salt stress, the expression patterns of *MsJAZs* under salt stresses were analyzed. Different *MsJAZs* exhibited different expression patterns under salt stress (Figure 8). Although most *MsJAZs* were upregulated, the time and degree of upregulation differed slightly. However, the same group of *MsJAZ* members showed similar trends. For example, group I members (*MsJAZ4, MsJAZ6, MsJAZ7, MsJAZ9*, and *MsJAZ11*) were all expressed instantaneously at 1 h or 2 h. Except for *MsJAZ9*, which had the highest expression level at 24 h, the expression of the remaining members gradually decreased to similar or slightly higher levels than at 0 h.

### 2.8. Analysis of Salt Resistance of Arabidopsis with Overexpression of MsJAZ1

**Figure 9:**
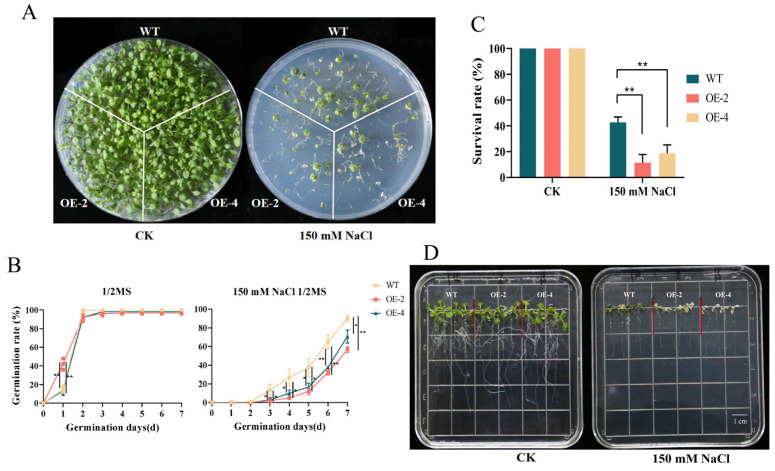
Analysis of salt resistance overexpressing *MsJAZ1* in *Arabidopsis*. (**A**–**C**) The growth of the WT and OE lines of *Arabidopsis* overexpressing *MsJAZ1* on a plate germinated for 20 days treated with CK and 150 mM NaCl. The statistical survival rate is shown in (**C**). The bottle green, orange, and yellow columns represent WT, OE-2, and OE-4, respectively. The asterisk on the column indicates a significant difference between WT and transgenic lines (* *p* < 0.05; ** *p* < 0.01, *t*-test). (**B**) Statistical analysis of the germination rate of *Arabidopsis* WT and OE lines overexpressing *MsJAZ1* from 0 to 7 days under CK and 150 mM NaCl treatment. The yellow, orange, and bottle green lines represent WT, OE-2, and OE-4, respectively. The asterisk on the column indicates significant difference between WT and transgenic lines (* *p* < 0.05; ** *p* < 0.01, *t*-test). (**D**) Salt tolerance analysis of WT and transgenic lines at the seedling stage.

Because *MsJAZ1* contains more stress-related *cis*-acting elements and has a significant response to salt stress, *MsJAZ1* was heterologously overexpressed in *Arabidopsis* to explore its function under salt stress. There was no significant difference in the survival rate of *Arabidopsis* between the WT and transgenic lines on 1/2 MS plates under normal conditions after 20 days of germination (Figure 9A,C). However, under 150 mM NaCl on 1/2 MS plates, the survival rate of WT was 42%, whereas the survival rates of OE-2 and OE-4 were only 11% and 18%, respectively, which were significantly lower than that of WT. The germination rates of the WT and transgenic lines were also explored. Under normal conditions, there was no significant difference in germination rates between the WT and transgenic lines OE-2 and OE-4 except on the second day (Figure 9B). However, under 150 mM NaCl treatment, the seed germination rate of the WT line was significantly faster than that of OE-2 and OE-4 from the third day. On the fourth day, WT was 5.65 and 2.6 times OE-2 and OE-4, respectively. On the seventh day, WT was 1.58 and 1.27 times more than OE-2 and OE-4, respectively. Moreover, when the seedlings grown normally for 7 days were placed on a 150 mM NaCl plate for 3 days, both OE-2 and OE-4 lines were albino (Figure 9D). Although WT was also partially albino, most of the leaves remained green. These results indicate that the overexpression of *MsJAZ1* in *Arabidopsis* makes *Arabidopsis* more sensitive to salt.

## 3. Discussion

JAZ is a crucial negative regulator of the JA signaling pathway. It plays a crucial role in plant growth and responses to both biotic and abiotic stresses [21,35]. Genome-wide identification of *JAZ* family members has been accomplished in many plants [10,33,36,37,38,39,40]. However, a comprehensive analysis and detailed study of the alfalfa *JAZ* family at the whole-genome level is incomplete. Therefore, we analyzed all *JAZ* genes in alfalfa to better understand the *JAZ* family at the molecular level and explore its resistance process.

A total of 11 *MsJAZ* genes were identified in this study, similar to those in *Arabidopsis*, suggesting a conserved evolutionary pattern among these species. This similarity may be attributed to using the “Zhongmu NO.1″ haploid reference genome [41]. Based on phylogenetic grouping information from *Arabidopsis* [42], the *JAZ* family members of *A. thaliana*, *M. sativa*, and *M. truncatula* were divided into five groups. Notably, group III did not contain any *JAZ* family members from alfalfa, suggesting that this species lacks a *JAZ* gene with a function similar to that of *AtJAZ10*. Exon–intron structures can provide further support for phylogenetic grouping [43]. In this study, most *MsJAZs* in the same group had similar gene structures, such as all group II members (*MsJAZ5, MsJAZ8*, and *MsJAZ10*) containing the same number of exons and similar lengths. However, there are cases in which individual members of the same group have different gene structures. For example, *MsJAZ11* in group I contains eight exons, which is different from other members, indicating that it may have functional divergences or new functions in the evolutionary process. Gene replication can also reflect the evolution of gene families and genomes [44]. In this study, five pairs of segmental repetition events were detected, among which the genes involved in the *MsJAZ5*/*MsJAZ8* and *MsJAZ5*/*MsJAZ10* gene pairs were all in the same clade. The genes involved in the *MsJAZ4*/*MsJAZ11* and *MsJAZ9*/*MsJAZ11* gene pairs were also in the same clade. The Ka/Ks ratios of the gene pairs suggested that the alfalfa *JAZ* gene family experienced purification selection during evolution, which might have resulted in preserved functionality or pseudogenization [40].

In recent years, there have been many studies on the functions of *JAZ* genes. In soybean, GmJAZ3 interacted with both GmRR18a and GmMYC2a, which can promote seed size, weight, and other organ sizes in stable transgenic soybean plants, increase the protein content of soybean seeds, and reduce the fatty acid content [45]. Through BLASTP, *MsJAZ1* and *MsJAZ3* were found to have high homology with *GmJAZ3*, reaching 88% and 73%, respectively. The expression levels of the two *MsJAZ* genes in leaves and elongating stems were higher than those in other tissues, so we speculated that *MsJAZ1* and *MsJAZ3* may affect the protein content of alfalfa by regulating the development of leaves and other tissues. Abiotic stresses seriously affect plant growth and productivity [46]. In this study, we found that all *MsJAZ* genes respond to salt stress, that different *MsJAZ* genes have various degrees of upregulation at different times, and that the expression patterns of members of the same clade are similar. Previous studies have shown that overexpression of *GhJAZ2* can significantly enhance the sensitivity of transgenic cotton to salt stress [47]. Rice salt sensitive 3 (RSS3) interacts with OsJAZ9, OsJAZ11, and non-R/B-like bHLH TFs to form a complex to regulate JA-mediated salt stress response [48]. In this study, *MsJAZ1* contained the largest number of stress-related cis-acting elements, and the qRT-PCR results showed that it responded to salt stress. Therefore, *MsJAZ1* was cloned and heterologously expressed in *A. thaliana* to further explore the function of *MsJAZ*. The results showed that under treatment with 150 mM NaCl, the germination and survival rates of transgenic *Arabidopsis* were significantly lower than those of the wild type (WT), and their overall growth was also poorer. These results suggest that *MsJAZ1* may negatively regulate plant salt tolerance.

Alfalfa is an important forage grass, but various adverse environments limit its growth. In recent years, with the development of various biotechnologies, breeding new varieties of alfalfa has become increasingly common. Therefore, this study provides a reference for further study on the function of the *MsJAZ* gene and the cultivation of new varieties with stress resistance.

## 4. Methods and Materials

### 4.1. Plant Materials and Growth Conditions

The alfalfa seeds “Zhongmu No. 1” were obtained from the Institute of Animal Science of the Chinese Academy of Agricultural Sciences. The growth conditions of the seedlings were consistent with the previous method [32]. Four weeks later, the seedlings were treated with 1/2 Hoagland solution containing 200 mM NaCl to simulate salt stress. The treatments were applied for 0, 1, 2, 4, 8, 12, and 24 hours (h), respectively, to mimic abiotic conditions. Every treatment had three biological replicates for each point, and all samples were stored at −80 °C for total RNA extraction.

### 4.2. Identification of JAZ Family Members in Alfalfa

The alfalfa reference genome sequence (Zhongmu No.1) was downloaded from Figshare (https://figshare.com/, accessed on 15 March 2023) [41]. The hidden Markov model (HMM) profile for the TIFY domain (PF06200) and Jas domain (PF09425) of the JAZ family were extracted from the Pfam database (https://www.ebi.ac.uk/interpro/entry/pfam/#table, accessed on 18 March 2023). Then, HMMER3.0 (http://hmmer.janelia.org/, accessed on 19 March 2023) was used to search alfalfa protein sequences that matched the JAZ HMM profiles, and sequences with an E-value less than 1.0 × 10^−5^ were selected. These candidates, *MsJAZ*, were submitted to NCBI-CDD (https://www.ncbi.nlm.nih.gov/cdd, accessed on 21 March 2023) to confirm further. The predicted isoelectric (pI) and molecular weights (MWs) of all *MsJAZ* genes were predicted with the ExPASy website (https://web.expasy.org/compute_pi/, accessed on 29 March 2023). Plant-mPLo (http://www.csbio.sjtu.edu.cn/bioinf/plant-multi/, accessed on 30 March 2023) was used to predict subcellular localization.

### 4.3. Chromosome Location Analysis and Phylogenetic Tree Construction

The genomic position of the identified *MsJAZs* was confirmed and mapped using the TBtools software (v1.108, Chen, C., GZ, China) [49]. JAZ family protein sequences in *A. thaliana* were downloaded from the TAIR website (https://www.arabidopsis.org/, accessed on 3 April 2023). *The JAZ* genes of *M. truncatula* were screened by BLASTp (https://blast.ncbi.nlm.nih.gov/Blast.cgi, accessed on 2 April 2023) and submitted to NCBI-CDD (https://www.ncbi.nlm.nih.gov/cdd, accessed on 2 April 2023). The JAZ protein sequences of *M. sativa*, *A. thaliana*, and *M. truncatula* were aligned using ClustalW with default parameters. A phylogenetic tree was then constructed using MEGA6.0 (Tamura, K., Tokyo, Japan), and the maximum likelihood method (bootstrap method with 1000 replications was selected) [50]. The iTOL website was used to beautify phylogenetic trees (https://itol.embl.de/, accessed on 6 April 2023) [51].

### 4.4. Gene Structure and Conserved Domains Analysis

The MEME website (https://meme-suite.org/meme/doc/meme.html, accessed on 8 April 2023) was used to analyze conserved motifs in MsJAZ proteins. The parameters in the program are selected: the maximum number of motifs was 10; the minimum was 6; the maximum motif width was 200 [52]. The gene structural information is included in the alfalfa gff file. Visualization using TBtools (v1.108, Chen, C., GZ, China) [35].

### 4.5. Cis-Acting Elements Analysis of MsJAZ Promoter

The upstream 2 kb sequence of the *MsJAZs* was extracted. Then, extracted sequences were submitted to the PlantCARE website (https://bioinformatics.psb.ugent.be/webtools/plantcare/html/, accessed on 13 April 2023) for *cis*-acting element analysis [53].

### 4.6. Gene Duplication and Synteny Analysis

Genomic data for *A. thaliana*, *M. truncatula*, and *G. max* were downloaded from the NCBI website (https://www.ncbi.nlm.nih.gov/, accessed on 25 April 2023). One-step MCScanX from TBtools (v1.108, Chen, C., GZ, China) with default parameters was used to analyze the pattern of gene duplication of *the JAZ* genes in *Medicago sativa.* Ka/Ks values for gene pairs were calculated using a simple Ka/Ks calculator in TBTools (v1.108, Chen, C., GZ, China). The Dual Synteny Plot in TBtools (v1.108, Chen, C., GZ, China) was used to determine the syntenic relationship between orthologous *MsJAZ* genes of alfalfa and other species.

### 4.7. Transcriptome Data Analysis

Transcriptomic data for six alfalfa tissues (leaves, nodules, elongating stems, flowers, post-elongation stems, and roots) were retrieved from the NCBI database (SRP055547) [54], and a correlation heatmap was analyzed using TBtools (v1.108, Chen, C., GZ, China) (Table S5).

### 4.8. Cloning and Expression Vector Construction of MsJAZ1

Total RNA from alfalfa leaves was extracted according to the instructions of the plant total RNA extraction kit (Promega, Madison, WI, USA), and cDNA was obtained by reverse transcription. The upstream primer MsJAZ1-F and downstream primer MsJAZ1-R were designed, and PCR amplification was performed using a cDNA template. The reaction was performed at 95 °C for 3 min. 95 °C, 30 s; 53 °C, 30 s; 72 °C, 1 min; 30 cycles; 72 °C for 5 min. A heterogenic expression vector was constructed, and the upstream primer MsJAZ1-eYFP-F and downstream primer MsJAZ1-eYFP-R were designed for amplification (Table S4). The amplified product was recovered and cloned seamlessly into the expression vector to obtain a recombinant plasmid. The correctly sequenced plasmid was transferred into *Agrobacterium tumefaciens* GV3101 (Huayueyang Biotechnology, Beijing, China) and transformed into *A. thaliana* (Cultivar “Col-0”) [55]. Transgenic plants were screened on 1/2 MS medium containing 4 mg/mL phosphinothricin (PPT) (Coolaber, Beijing, China) for PCR detection. Transgenic *A. thaliana* was planted by a single plant until homozygous plants were obtained.

### 4.9. Detection of Salt Tolerance in Transgenic Arabidopsis

Seeds of homozygous lines of transgenic and wild-type plants were disinfected and seeded on 1/2 MS and 150 mM NaCl 1/2 MS, respectively. The germination rate was counted every day from 0 to 7. The phenotype was observed, and the survival rate was calculated on the 20th day. The seedlings growing normally for 7 days were cultured in 1/2 MS and 150 mM NaCl 1/2 MS for 3 days, respectively. Each medium contained four *Arabidopsis* seedlings. Each experiment was performed in triplicates.

### 4.10. Real-Time Quantitative PCR (RT-qPCR) Analysis

The reagents, instruments, and methods used in RT-qPCR were consistent with the previous methods [56]. RT-qPCR-specific primers for 11 *MsJAZ* genes were designed using the Primer5.0 software (Premierbiosoft, San Francisco, USA). Primer sequences are shown in Table S3. The data from three biological replicates and three technical replicates were analyzed statistically. RT-qPCR was used to analyze the relative gene expression levels using the 2^−∆∆Ct^ method.

### 4.11. Statistical Analysis

SPSS 25 software (IBM Inc., Chicago, IL, USA) was used for statistical analysis. The Duncan multiple range test was used to analyze differences between groups (ANOVA with *p* < 0.05). GraphPad Prism 8.0.2 software (GraphPad Software, Boston, FL, USA) was used to plot the data. Adobe Illustrator CC 2020 (ADOBE, San Jose, CA, USA) was used for graphic editing.

## 5. Conclusions

In this study, the *JAZ* gene family members of *M. sativa* were analyzed using bioinformatics tools, and the function of *MsJAZ1* under salt stress was explored. A total of 11 *MsJAZ* genes were identified, which were distributed on five chromosomes. The phylogenetic tree divided *MsJAZs* into five groups. The members of each branch contained similar gene structures and motifs, indicating that members of the same branch may contain similar functions. Segmental replication was the main driving force for evolution in the *MsJAZs* gene family. During evolution, the *MsJAZ* gene family has experienced purification selection. The expression pattern of the *MsJAZ* gene in six different tissues of alfalfa showed that 45% of *MsJAZ* genes were highly expressed in leaves, and some genes were highly expressed in roots and elongating stems. RT-qPCR results showed that all *MsJAZ* genes responded to salt stress. Under salt stress, the germination rate and survival rate of overexpressing *MsJAZ1 Arabidopsis* were significantly decreased, affecting seedling growth, indicating that *MsJAZ1* negatively regulates plant salt tolerance. In summary, the results of this study lay a foundation for further exploration of the function of *MsJAZs*.

## Figures and Tables

**Table 1:** Characteristics of *MsJAZ* genes in *Medicago sativa*.

Gene Name	Gene ID	Full CDS Length (bp)		Protein		Subcellular Location
			Length (aa)	Mw (kDa)	pI	
*MsJAZ1*	MsG0180001147	984	327	34.29	9.45	Cell membrane nucleus
*MsJAZ2*	MsG0280007019	396	131	15.09	8.86	Nucleus
*MsJAZ3*	MsG0280007633	888	295	31.11	7.10	Cell membrane nucleus
*MsJAZ4*	MsG0280008436	753	250	27.15	8.83	Nucleus
*MsJAZ5*	MsG0280008590	627	208	22.36	8.41	Nucleus
*MsJAZ6*	MsG0580024589	837	278	31.71	9.56	Nucleus
*MsJAZ7*	MsG0580024590	588	195	22.81	8.42	Nucleus
*MsJAZ8*	MsG0680032555	630	209	22.41	8.52	Nucleus
*MsJAZ9*	MsG0880042776	708	235	25.91	8.34	Nucleus
*MsJAZ10*	MsG0880047288	537	178	19.33	8.97	Nucleus
*MsJAZ11*	MsG0880047330	1014	337	38.03	8.34	Nucleus

CDS: coding sequence; bp: base pair; aa: amino acid; MW: molecular weight; pI: isoelectric point.

**Table 2:** Ks and Ka analysis of duplicated gene pairs.

Gene1	Gene2	Ka	Ks	Ka/Ks	Duplication Type
*MsJAZ4*	*MsJAZ9*	0.643651984	1.384563729	0.46487711	Segmental duplication
*MsJAZ4*	*MsJAZ11*	0.317998017	0.795918567	0.39953587	Segmental duplication
*MsJAZ5*	*MsJAZ8*	0.01802844	0.038425167	0.469183122	Segmental duplication
*MsJAZ5*	*MsJAZ10*	0.394218786	0.584020786	0.675008142	Segmental duplication
*MsJAZ9*	*MsJAZ11*	0.608088468	2.200562393	0.276333209	Segmental duplication

Ka: Non-synonymous substitution rate. Ks: Synonymous substitution rate.

## Data Availability

Data are contained within the article and Supplementary Materials.

## Supplementary Materials

The supporting information can be downloaded at: https://www.mdpi.com/article/10.3390/ijms251910589/s1.
