# Supplementary material for: Genome-Wide Identification, Phylogenetic, and Expression Analysis of Jasmonate ZIM-Domain Gene Family in Medicago Sativa L
Source: Int J Mol Sci. 2024 Oct 1;25(19):10589. doi: 10.3390/ijms251910589 (PMC11477025; doi:10.3390/ijms251910589)
Supplement: Supplementary file 1 [file ijms-25-10589-s001.zip › Figure S1.pdf]

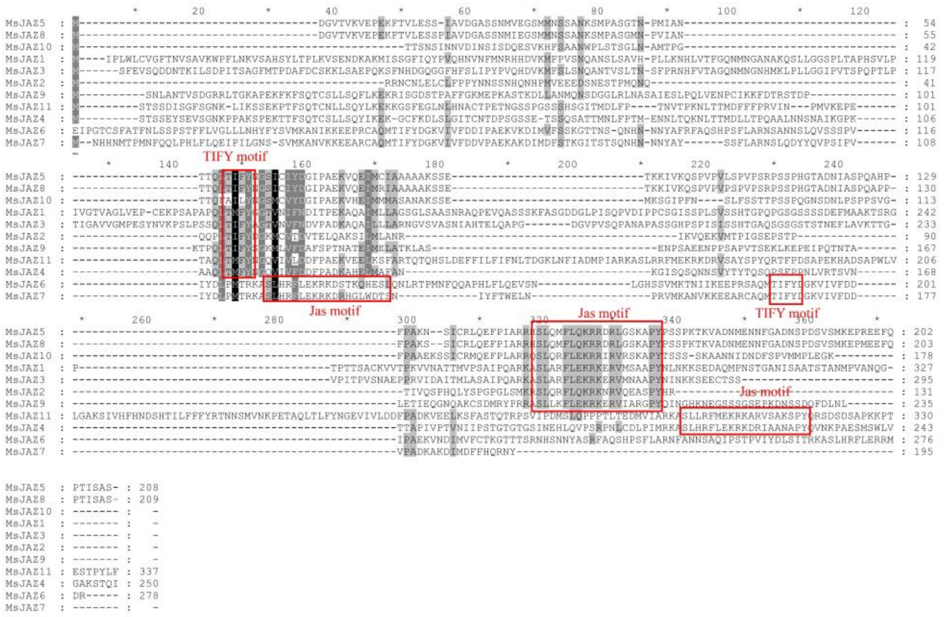

Figure S1. **Multiple sequence alignment of the MsJAZ proteins.** The TIFY and JAZ regions are indicated by red boxes.
